# Supplementary material for: Cardiometabolic Differences in People Living with HIV Receiving Integrase Strand Transfer Inhibitors Compared to Non-nucleoside Reverse Transcriptase Inhibitors: Implications for Current ART Strategies
Source: Viruses. 2024 Apr 10;16(4):582. doi: 10.3390/v16040582 (PMC11054623; doi:10.3390/v16040582)
Supplement: Supplementary file 1 [file viruses-16-00582-s001.zip › viruses-2931554-supplementary.pdf]

# Supplementary material

Supplementary table S1: Table of intercorrelated lipoproteins and lipids and their cluster representatives

| Cluster no | Cluster representative | Lipoproteins in cluster |
|---|---|---|
| 1 | **M_LDL_C** | ApoB<br>L_LDL_P<br>LDL_CE<br>LDL_L<br>LDL_P<br>LDL_PL<br>M_LDL_CE<br>M_LDL_L<br>M_LDL_P<br>M_LDL_PL<br>M_VLDL_C<br>M_VLDL_CE<br>non_HDL_C<br>S_LDL_C<br>S_LDL_CE<br>S_LDL_L<br>S_LDL_P<br>S_LDL_PL<br>M_LDL_C |
| 2 | **XS_VLDL_C** | IDL_P<br>Remnant_C<br>XS_VLDL_CE<br>XS_VLDL_FC<br>XS_VLDL_L<br>XS_VLDL_P<br>XS_VLDL_PL<br>XS_VLDL_C |
| 3 | **L_VLDL_C** | L_VLDL_CE<br>M_VLDL_FC<br>M_VLDL_L<br>M_VLDL_P<br>M_VLDL_PL<br>S_VLDL_C<br>S_VLDL_CE<br>S_VLDL_FC<br>S_VLDL_L<br>S_VLDL_P<br>S_VLDL_PL<br>VLDL_C<br>VLDL_CE<br>VLDL_FC<br>VLDL_L<br>VLDL_P<br>VLDL_PL<br>XL_VLDL_C<br>XL_VLDL_CE<br>L_VLDL_C |
| 4 | **LDL_TG** | HDL_TG<br>IDL_TG<br>L_LDL_TG<br>M_HDL_TG<br>M_LDL_TG<br>MUFA<br>SFA<br>Total_FA<br>XS_VLDL_TG<br>LDL_TG |
| 5 | **Total_PL** | LA<br>Omega_6<br>PUFA<br>Total_L<br>Total_PL |
| 6 | **IDL_C** | Clinical_LDL_C<br>IDL_CE<br>IDL_FC<br>IDL_L<br>IDL_PL<br>L_LDL_C<br>L_LDL_CE<br>L_LDL_FC<br>L_LDL_L<br>L_LDL_PL<br>LDL_FC<br>M_LDL_FC<br>Total_C<br>Total_CE<br>Total_FC<br>IDL_C |
| 7 | **HDL_C** | ApoA1<br>HDL_CE<br>HDL_FC<br>HDL_L<br>HDL_P<br>HDL_PL<br>M_HDL_C<br>M_HDL_CE<br>M_HDL_FC<br>M_HDL_L<br>M_HDL_P<br>Total_P<br>HDL_C |
| 8 | **L_HDL_C** | HDL_size<br>L_HDL_CE<br>L_HDL_FC<br>L_HDL_L<br>L_HDL_P<br>L_HDL_PL<br>L_HDL_C |
| 9 | **VLDL_TG** | L_VLDL_FC<br>L_VLDL_L<br>L_VLDL_P<br>L_VLDL_PL<br>L_VLDL_TG<br>M_VLDL_TG<br>S_HDL_TG<br>S_LDL_TG<br>S_VLDL_TG<br>Total_TG<br>VLDL_size<br>XL_VLDL_FC<br>XL_VLDL_L<br>XL_VLDL_P<br>XL_VLDL_PL<br>XL_VLDL_TG<br>XXL_VLDL_C<br>XXL_VLDL_CE<br>XXL_VLDL_FC<br>XXL_VLDL_L<br>XXL_VLDL_P<br>XXL_VLDL_PL<br>VLDL_TG |
| 10 | **XL_HDL_C** | XL_HDL_CE<br>XL_HDL_L<br>XL_HDL_P<br>XL_HDL_PL<br>XL_HDL_C |
| 11 | **S_HDL_C** | S_HDL_FC<br>S_HDL_L<br>S_HDL_P<br>S_HDL_PL<br>S_HDL_C |
| 12 | **Omega_3** | Omega_3 |

Supplementary figure S1: Principal component analysis of untargeted metabolomics. Two samples had were identified as outliers (SD>4) and removed from further analysis.

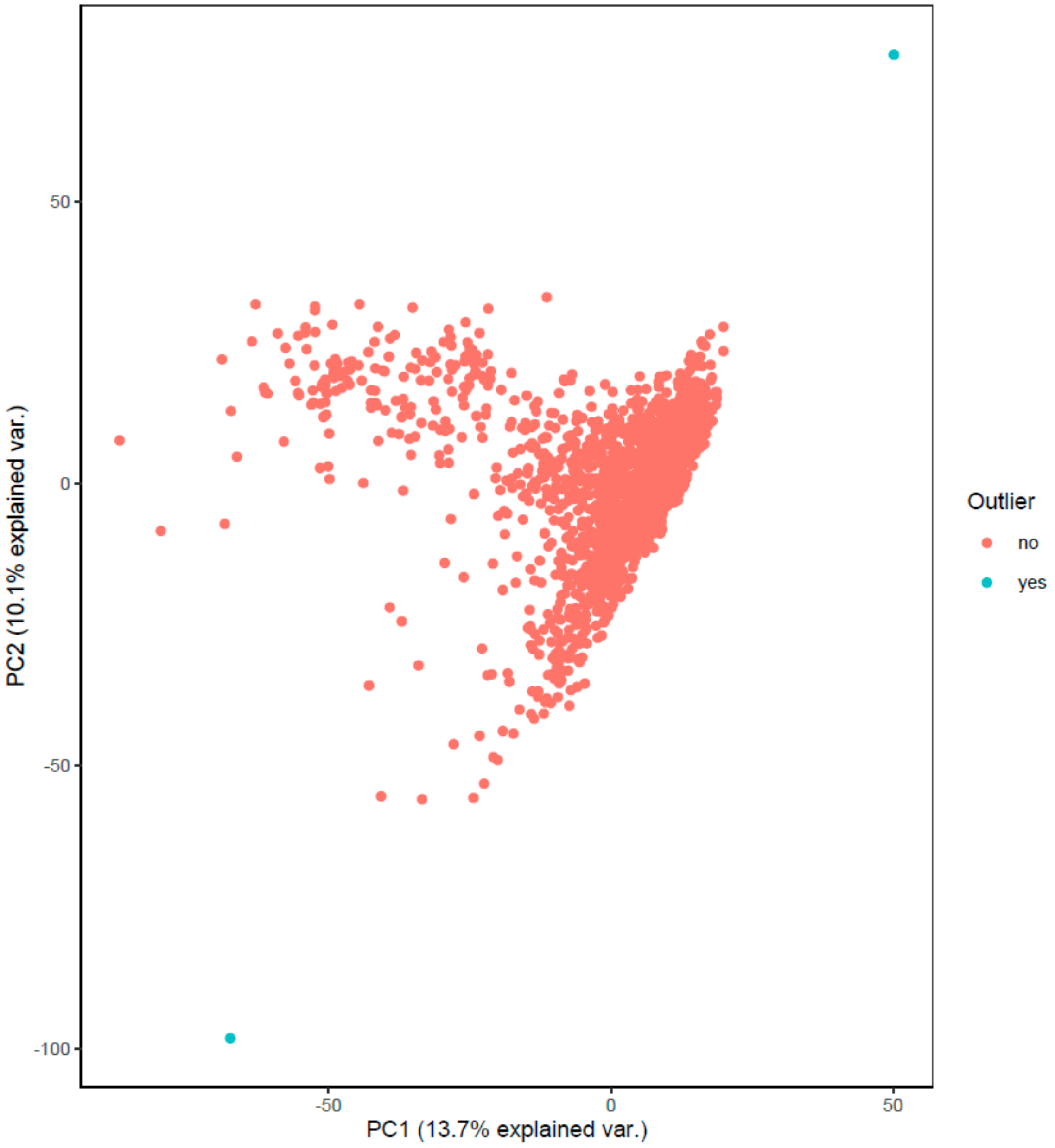

Supplementary figure S2: Contributions of the different principal components (PC) to explain differences in metabolite levels in the 2000HIV cohort. Only PC1 and PC2 had an influence of >5% on metabolite levels.

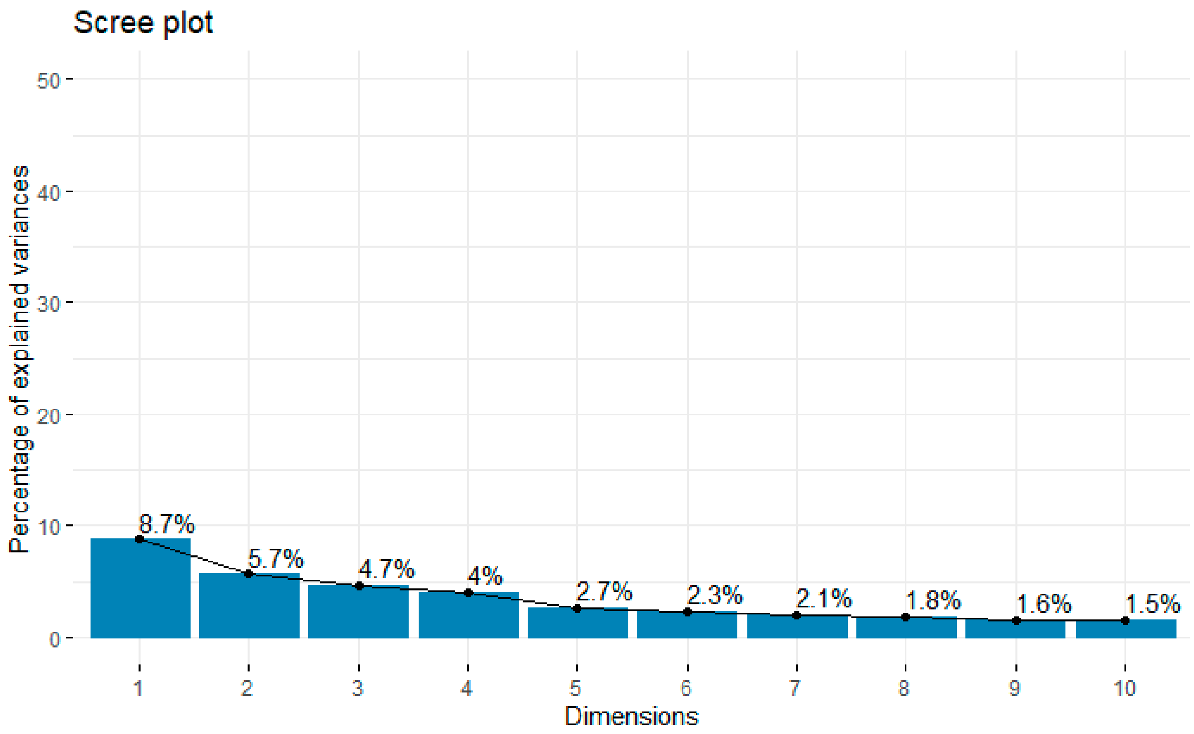

<u>Supplementary figure S3:</u> Principal component analysis of metabolite levels. Only age appears to have confounding influence on metabolite levels.

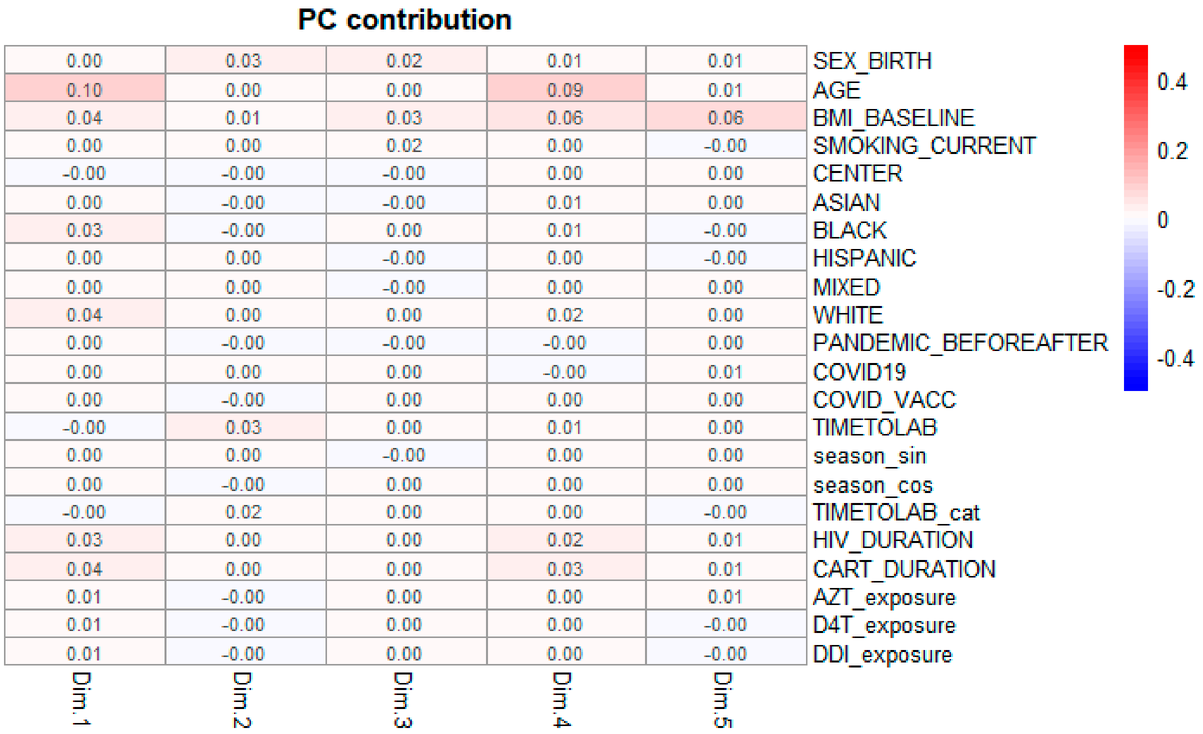


Explanation of variables: SEX_BIRTH: sex at birth; CENTER: site where participant participated in the study; MIXED: of mixed ethnicity; PANDEMIC_BEFOREAFTER: participant participated before or after the onset of the covid-19 pandemic in the Netherlands (march 2020); COVID19: did participant have COVID-19 before participation; COVID_VACC: was participant vaccinated for COVID-19 before participation; season_cos/season_sin: seasonality influence of metabolite levels; TIMETOLAB_cat: categorical varible of time between sample draw and sample processing (< vs > 24hours);

Supplementary figure S4: Participant selection for analysis from 2000HIV study. For the current analysis we considered all participants in the 2000HIV study and selected only participants not using immunomodulatory medication and those using one or two N(t)RTI's in combination with either one INSTI or one NNRTI. Previously, all participants were already divided into a discovery and validation cohort based on their site of participation.

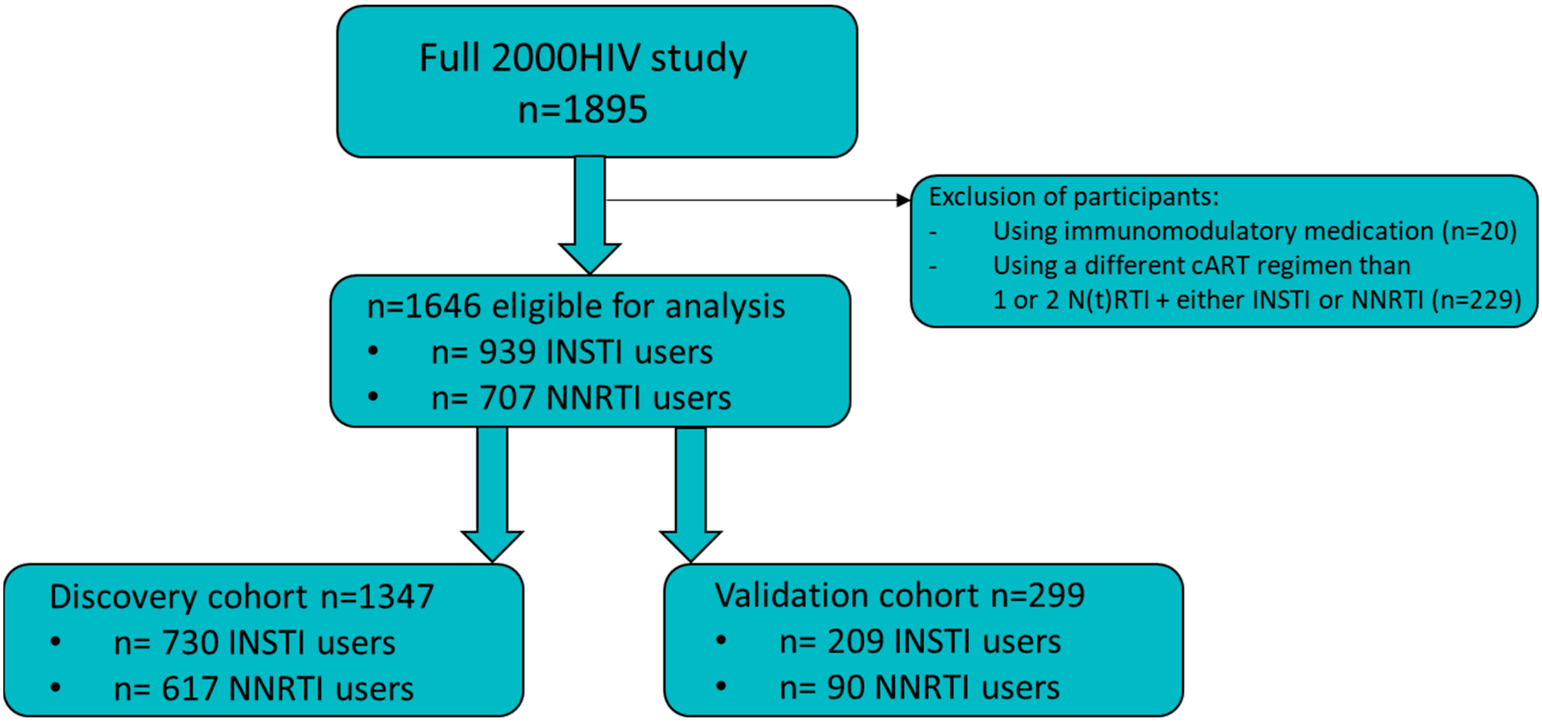

Supplementary figure S5: Type of INSTI and NNRTI in use in discovery and validation cohort.

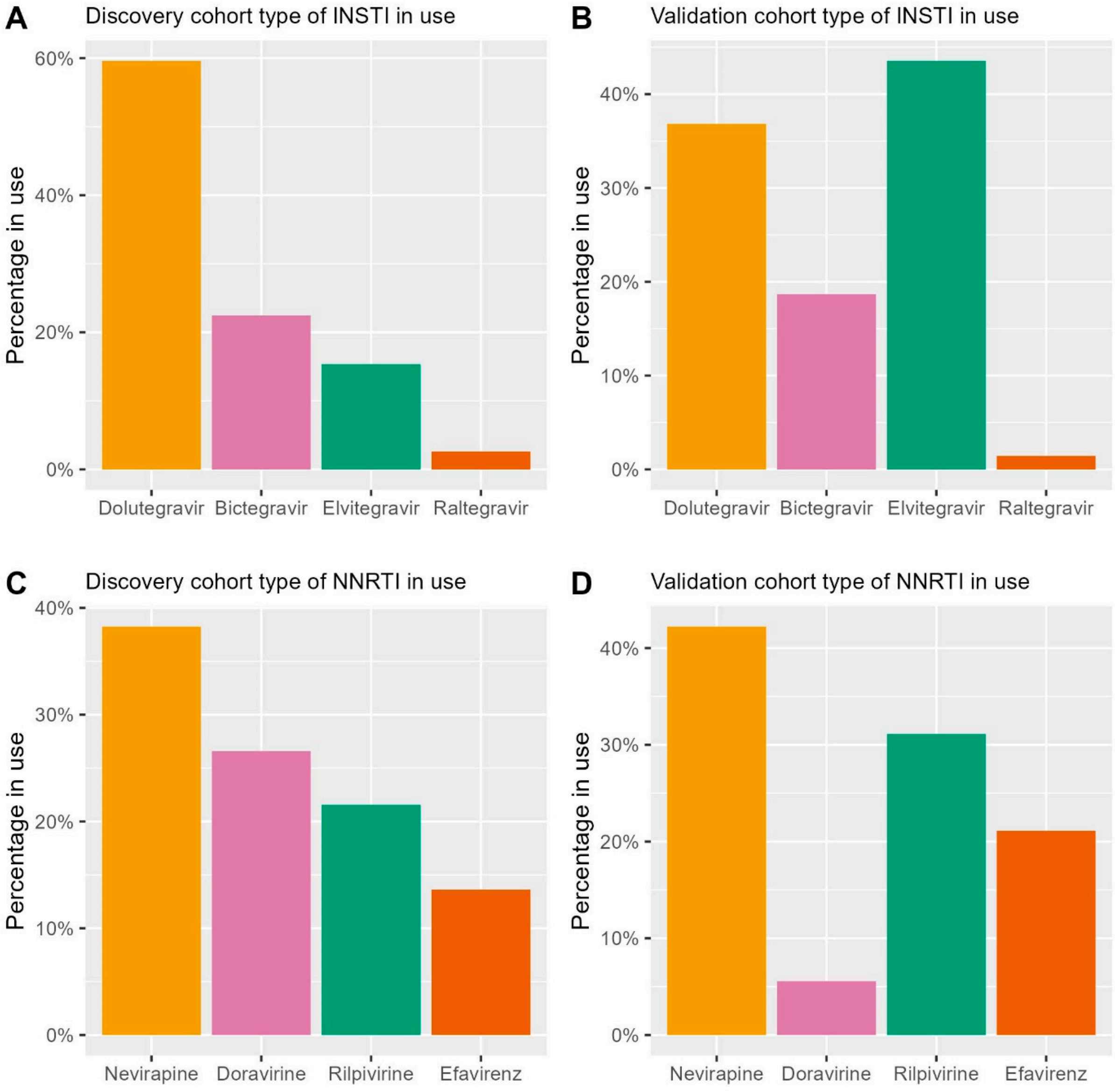

Supplementary figure S6: Principal component (PC) analysis of untargeted plasma metabolites of the types of INSTI and NNRTI in the discovery cohort. X-axis shows PC1, Y-axis shows PC2.

A: All types of INSTI showed overlap in PC analysis, showing homogeneity between untargeted plasma metabolomics between the different INSTI types.

B: All types of NNRTI showed overlap in PC analysis, showing homogeneity between untargeted plasma metabolomics between the different NNRTI types.

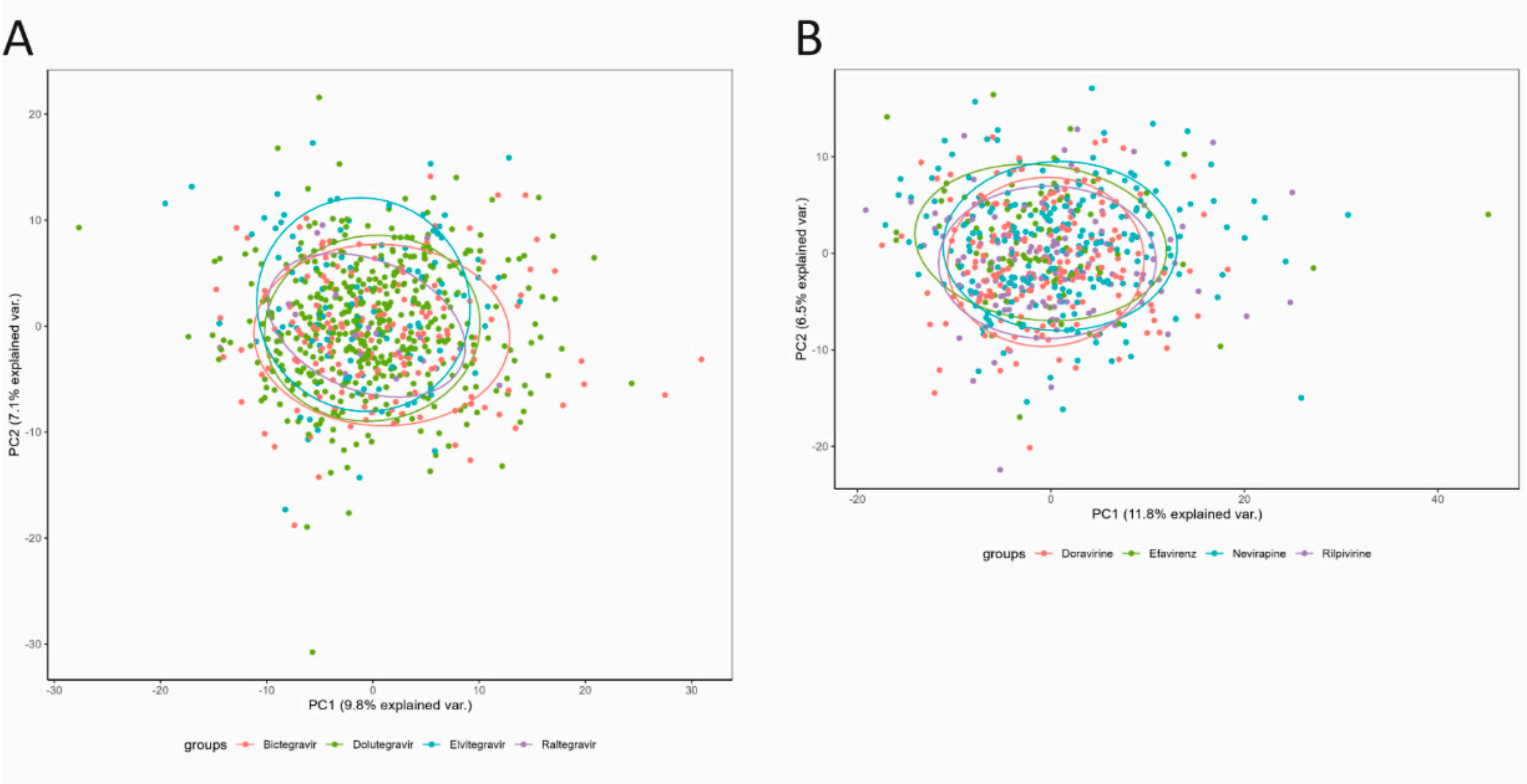

Supplementary figure S7: Differentially expressed untargeted metabolites in INSTI users compared to NNRTI users in the validation cohort (INSTI n = 207; NNRTI n = 89). Differential expression analysis using a linear model with sex at birth and age as covariates on 500 metabolites known to be present in serum. Y-axis shows the p-value through -log10(p-value), x-axis shows the log fold change. Horizontal dotted line represents border of significance (p-value<0.05), vertical dotted line represents border between higher levels (right) and lower levels (left) of metabolites in the INSTI group. Significantly differentially expressed metabolites are shown in red. Numbers specify the significant differentially expressed ion m/z ratio. In the validation cohort cohort, 32 metabolites were significantly higher and 39 metabolites were significantly lower in the INSTI group. In addition, 283 metabolites had a positive log fold change and 217 metabolites had a negative log fold change.

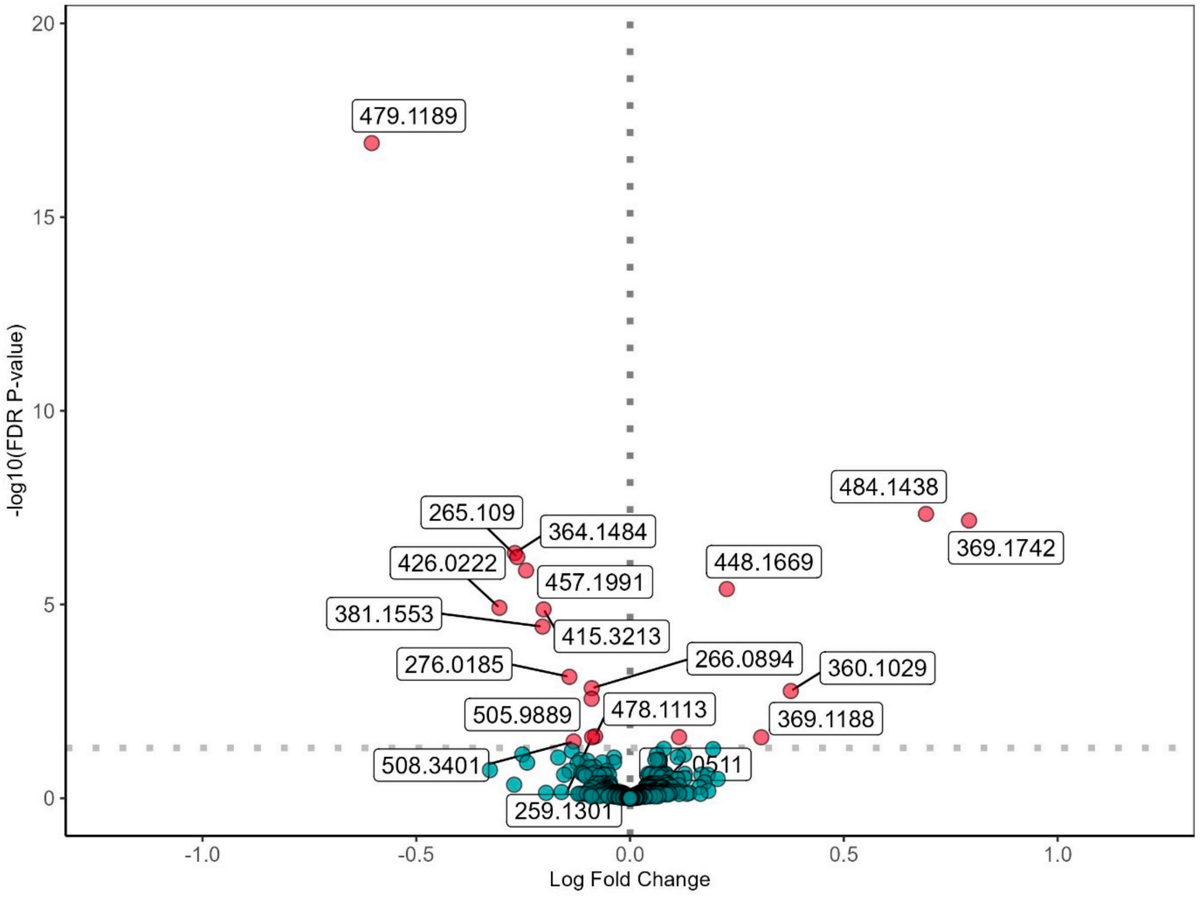

Supplementary table S2: Overlapping significant differentially expressed metabolites in INSTI compared to NNRTI users with metabolite annotations in discovery and validation cohort with FDR/P-values and log fold changes. Metabolites with identical ionMz share colors.

| ionMz | name | logfc discovery | fdr discovery | logfc validation | pvalue validation |
|---|---|---|---|---|---|
| 457.1362 | 12-Hydroxynevirapine glucuronide | -1,170858737 | 7,77581E-72 | -1,294790654 | 6,34292E-23 |
| 457.1362 | 2-Hydroxynevirapine glucuronide | -1,170858737 | 7,77581E-72 | -1,294790654 | 6,34292E-23 |
| 457.1362 | 3-Hydroxynevirapine glucuronide | -1,170858737 | 7,77581E-72 | -1,294790654 | 6,34292E-23 |
| 457.1362 | 8-Hydroxynevirapine glucuronide | -1,170858737 | 7,77581E-72 | -1,294790654 | 6,34292E-23 |
| 479.1189 | 3'-O-Methylepicatechin 7-O-glucuronide | -0,554835062 | 4,3495E-63 | -0,60426381 | 4,92754E-20 |
| 479.1189 | 4'-O-Methyl-(-)-epicatechin 3'-O-glucuronide | -0,554835062 | 4,3495E-63 | -0,60426381 | 4,92754E-20 |
| 369.1742 | Androsterone sulfate | 0,743880297 | 1,60521E-38 | 0,792810872 | 5,42996E-10 |
| 369.1742 | 5a-Dihydrotestosterone sulfate | 0,743880297 | 1,60521E-38 | 0,792810872 | 5,42996E-10 |
| 369.1742 | Epiandrosterone sulfate | 0,743880297 | 1,60521E-38 | 0,792810872 | 5,42996E-10 |
| 448.1669 | Glisoxepide | 0,211828559 | 1,07168E-30 | 0,226176781 | 6,38694E-08 |
| 251.1081 | Cimetidine | -0,134399869 | 3,45922E-24 | -0,116438234 | 0,006122943 |
| 364.1484 | Propericiazine | -0,218339292 | 4,21302E-20 | -0,269504059 | 4,69445E-09 |
| 484.1438 | Ketotifen-N-glucuronide | 0,274942201 | 3,0648E-19 | 0,692051409 | 2,75413E-10 |
| 265.109 | Nevirapine | -0,180694241 | 3,77874E-17 | -0,263818024 | 7,17281E-09 |
| 457.1991 | 2-Hydroxy-desipramine glucuronide | -0,184537655 | 4,13818E-17 | -0,243330316 | 1,85236E-08 |
| 112.0511 | Creatinine | 0,109513658 | 2,75081E-12 | 0,114711351 | 0,000897632 |
| 167.0212 | Uric acid | 0,145224431 | 2,96648E-12 | 0,126667806 | 0,003866144 |
| 381.1553 | Ibuprofen glucuronide | -0,149986012 | 2,78661E-11 | -0,204637832 | 8,17831E-07 |
| 462.0555 | Chondroitin sulfate | -0,075592181 | 1,47488E-09 | -0,064914894 | 0,008556359 |
| 315.0511 | Isorhamnetin | -0,03467816 | 1,66986E-09 | -0,037406184 | 0,005122368 |
| 383.1532 | 16alpha-Hydroxy DHEA 3-sulfate | -0,252454815 | 9,07659E-09 | -0,251970071 | 0,003558627 |
| 266.0894 | Adenosine | -0,045440073 | 2,24795E-05 | -0,089556921 | 3,75408E-05 |
| 266.0894 | Deoxyguanosine | -0,045440073 | 2,24795E-05 | -0,089556921 | 3,75408E-05 |
| 266.0894 | Vidarabine | -0,045440073 | 2,24795E-05 | -0,089556921 | 3,75408E-05 |
| 266.0894 | Zidovudine | -0,045440073 | 2,24795E-05 | -0,089556921 | 3,75408E-05 |
| 360.1029 | 5-Hydroxyomeprazole | 0,157370187 | 3,20968E-05 | 0,375888271 | 4,78265E-05 |
| 360.1029 | Omeprazole sulfone | 0,157370187 | 3,20968E-05 | 0,375888271 | 4,78265E-05 |
| 360.1029 | 3-Hydroxyomeprazole | 0,157370187 | 3,20968E-05 | 0,375888271 | 4,78265E-05 |
| 427.2251 | Irbesartan | 0,154868448 | 4,20224E-05 | 0,194148894 | 0,002362308 |
| 128.0349 | Pyroglutamic acid | -0,07251005 | 0,000140932 | -0,089274319 | 0,025839989 |
| 128.0349 | dimethadione | -0,07251005 | 0,000140932 | -0,089274319 | 0,025839989 |
| 367.1588 | Dehydroepiandrosterone sulfate | 0,195192514 | 0,000228188 | 0,205008978 | 0,045121886 |
| 367.1588 | Testosterone sulfate | 0,195192514 | 0,000228188 | 0,205008978 | 0,045121886 |
| 146.0456 | L-Glutamic acid | -0,083316051 | 0,00024846 | -0,136087126 | 0,002754033 |
| 146.0456 | O-Acetylserine | -0,083316051 | 0,00024846 | -0,136087126 | 0,002754033 |
| 146.0456 | D-Glutamic acid | -0,083316051 | 0,00024846 | -0,136087126 | 0,002754033 |
| 146.0456 | DL-Glutamate | -0,083316051 | 0,00024846 | -0,136087126 | 0,002754033 |
| 183.139 | Undecylenic acid | 0,07609794 | 0,000359433 | 0,111445782 | 0,004655494 |
| 164.072 | L-Phenylalanine | 0,037448861 | 0,000725874 | 0,059139569 | 0,006770527 |
| 350.0279 | Meloxicam | -0,082092378 | 0,000843835 | -0,122548288 | 0,009334358 |

| ionMz | name | logfc discovery | fdr discovery | logfc validation | pvalue validatior |
|---|---|---|---|---|---|
| 224.0785 | Aciclovir | 0,09748719 | 0,001052967 | 0,127764519 | 0,023955153 |
| 478.1113 | Petunidin 3-glucoside | -0,039701752 | 0,001990301 | -0,081900186 | 0,000805794 |
| 426.0222 | ADP | -0,090240251 | 0,002206316 | -0,305559259 | 2,18393E-07 |
| 102.0555 | Dimethylglycine | -0,051815557 | 0,003356823 | -0,072853297 | 0,034513634 |
| 102.0555 | gamma-Aminobutyric acid | -0,051815557 | 0,003356823 | -0,072853297 | 0,034513634 |
| 102.0555 | L-alpha-Aminobutyric acid | -0,051815557 | 0,003356823 | -0,072853297 | 0,034513634 |
| 102.0555 | D-alpha-Aminobutyric acid | -0,051815557 | 0,003356823 | -0,072853297 | 0,034513634 |
| 102.0555 | 2-Aminoisobutyric acid | -0,051815557 | 0,003356823 | -0,072853297 | 0,034513634 |
| 102.0555 | (S)-beta-Aminoisobutyric acid | -0,051815557 | 0,003356823 | -0,072853297 | 0,034513634 |
| 102.0555 | 3-Aminoisobutanoic acid | -0,051815557 | 0,003356823 | -0,072853297 | 0,034513634 |
| 96.9592 | Sulfate | 0,031318802 | 0,005163462 | 0,062582775 | 0,00391384 |
| 203.0827 | L-Tryptophan | 0,048379387 | 0,005332126 | 0,063383446 | 0,031678514 |
| 203.0827 | D-Tryptophan | 0,048379387 | 0,005332126 | 0,063383446 | 0,031678514 |
| 203.0827 | 3-Hydroxymethylantipyrine | 0,048379387 | 0,005332126 | 0,063383446 | 0,031678514 |
| 203.0827 | Ethotoin | 0,048379387 | 0,005332126 | 0,063383446 | 0,031678514 |
| 203.0827 | Nirvanol | 0,048379387 | 0,005332126 | 0,063383446 | 0,031678514 |
| 203.0827 | 4-Hydroxyantipyrine | 0,048379387 | 0,005332126 | 0,063383446 | 0,031678514 |
| 203.0827 | S-nirvanol | 0,048379387 | 0,005332126 | 0,063383446 | 0,031678514 |
| 195.0654 | Homoveratric acid | -0,099877343 | 0,005392802 | -0,154365506 | 0,030736962 |
| 195.0654 | 3-(3-Hydroxyphenyl)-2-methyllactic acid | -0,099877343 | 0,005392802 | -0,154365506 | 0,030736962 |
| 195.0654 | 3-(3,4-Dihydroxyphenyl)-2-methylpropionic aci | -0,099877343 | 0,005392802 | -0,154365506 | 0,030736962 |
| 195.0654 | Dihydroferulic acid | -0,099877343 | 0,005392802 | -0,154365506 | 0,030736962 |
| 195.0654 | 3-(3-hydroxy-4-methoxyphenyl)propanoic acid | -0,099877343 | 0,005392802 | -0,154365506 | 0,030736962 |
| 276.0185 | DOPA sulfate | -0,0590246 | 0,005392802 | -0,141979154 | 1,76683E-05 |
| 415.3213 | 24,25-Dihydroxyvitamin D | -0,054332113 | 0,01042519 | -0,202276475 | 2,66684E-07 |
| 415.3213 | 25,26-dihydroxyvitamin D | -0,054332113 | 0,01042519 | -0,202276475 | 2,66684E-07 |
| 415.3213 | Calcitriol | -0,054332113 | 0,01042519 | -0,202276475 | 2,66684E-07 |
| 415.3213 | 3beta-Hydroxy-5-cholestenoic acid | -0,054332113 | 0,01042519 | -0,202276475 | 2,66684E-07 |
| 415.3213 | Paricalcitol | -0,054332113 | 0,01042519 | -0,202276475 | 2,66684E-07 |
| 259.1301 | gamma-Glutamylisoleucine | -0,034999121 | 0,010792483 | -0,088837457 | 0,001015475 |
| 259.1301 | gamma-Glutamylleucine | -0,034999121 | 0,010792483 | -0,088837457 | 0,001015475 |
| 182.0463 | 4-Pyridoxic acid | -0,080798755 | 0,011311974 | -0,108927018 | 0,023094951 |
| 195.0521 | 1,3-Dimethyluric acid | -0,120179041 | 0,019570213 | -0,240823458 | 0,008913258 |
| 195.0521 | 3,7-Dimethyluric acid | -0,120179041 | 0,019570213 | -0,240823458 | 0,008913258 |
| 195.0521 | 1,9-Dimethyluric acid | -0,120179041 | 0,019570213 | -0,240823458 | 0,008913258 |
| 195.0521 | 7,9-Dimethyluric acid | -0,120179041 | 0,019570213 | -0,240823458 | 0,008913258 |
| 195.0521 | 1,7-Dimethyluric acid | -0,120179041 | 0,019570213 | -0,240823458 | 0,008913258 |
| 508.3401 | LysoPE(20:0/0:0) | -0,051859038 | 0,027230985 | -0,131899344 | 0,001370067 |
| 508.3401 | LysoPC(17:0/0:0) | -0,051859038 | 0,027230985 | -0,131899344 | 0,001370067 |
| 116.0502 | Indole | 0,034202513 | 0,035415296 | 0,060391926 | 0,0284867 |

Supplementary figure S8: Comparison of untargeted metabolites found in the discovery and validation cohort. Circles indicate number of metabolites found in certain category. Overlaying parts of the circles indicate number of metabolites found to overlap between the two categories. A: Overlap between FDR significant higher levels of metabolites in discovery cohort and p-value significant higher levels of metabolites in validation cohort. B: Overlap between FDR significant lower levels of metabolites in discovery cohort and p-value significant lower levels of metabolites in validation cohort. C: Overlap between FDR significant higher levels of metabolites in discovery cohort and metabolites with a positive fold change in the validation cohort. D: Overlap between FDR significant lower levels of metabolites in discovery cohort and metabolites with a negative fold change in the validation cohort.

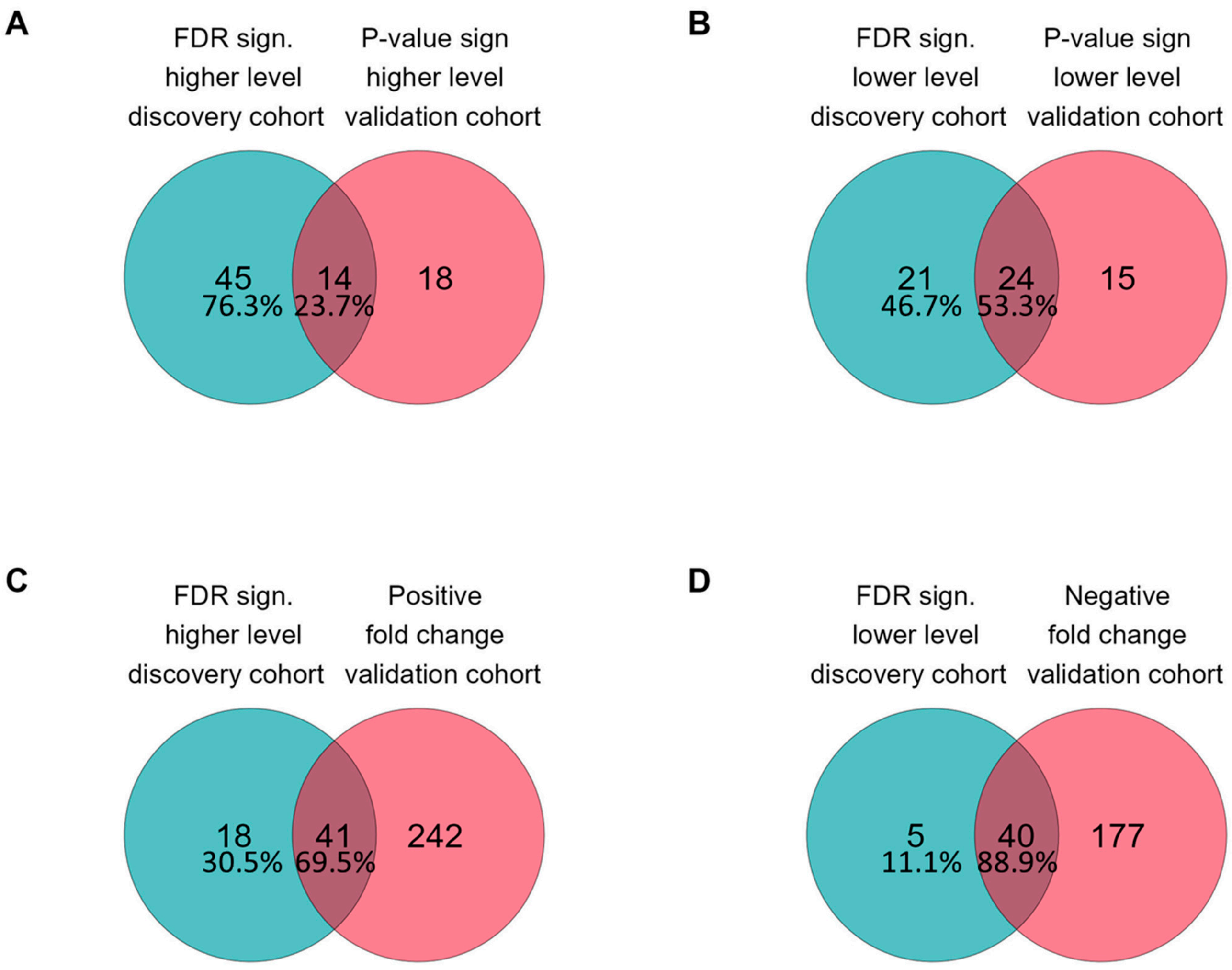

<u>Supplementary figure S9:</u> Up- and down-regulation of metabolic pathways in INSTI users compared to NNRTI users in the validation cohort (INSTI n = 207; NNRTI n = 89) using MetaboAnalyst software. Y-axis shows significance through -10log(P-value), with the horizontal line the threshold for significance ($p<0.05$). X-axis indicates normalized enrichment score (NES) with horizontal line represents border between upregulated (right side) and downregulated (left side). Red dots indicate significant pathways. In the validation cohort nine pathways were significantly downregulated, three pathways were upregulated in INSTI users compared to NNRTI users. In addition, the three pathways that were significantly differentially regulated in the discovery cohort and had same directionality in the validation cohort without reaching the threshold of significance were annotated.

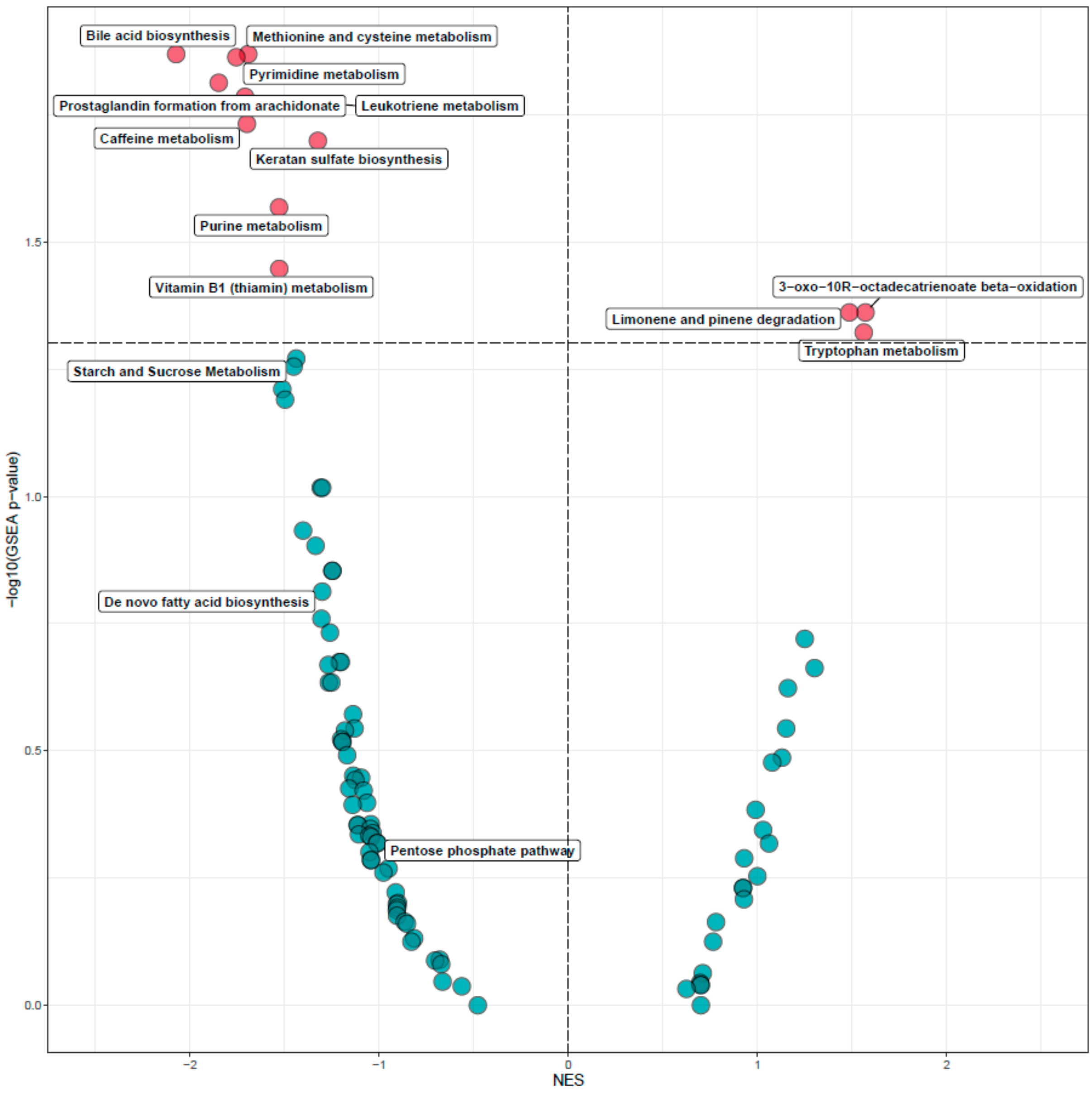

Supplementary figure S10: Nonhierarchical clustering of 132 intercorrelated lipoproteins and lipids through hierarchical Ward-linkage clustering based on Spearman correlation coefficients. Clusters share the same color, all clusters were positively correlated (r>0.75, p<0.05).

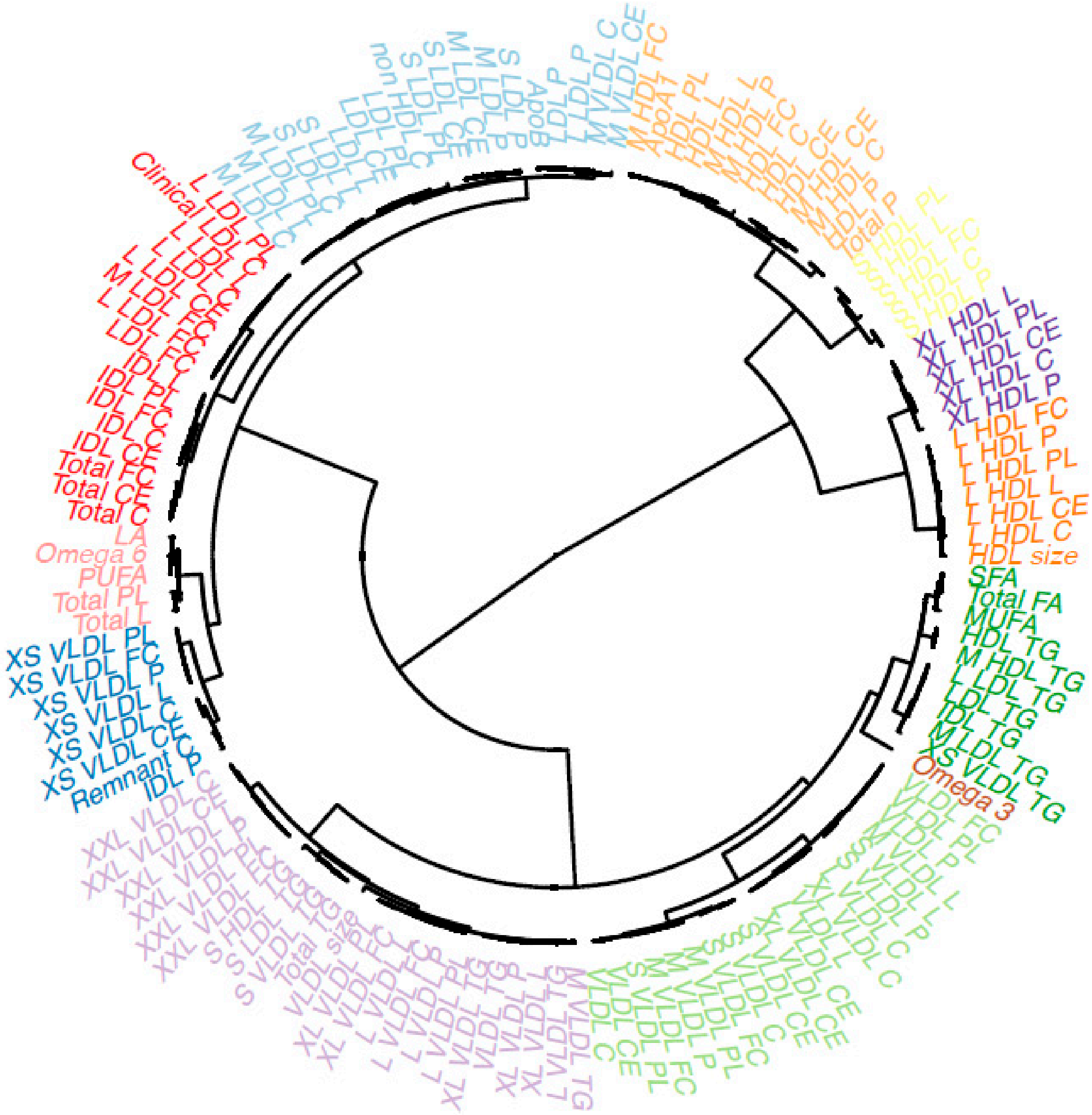

Supplementary figure S11: Principal component (PC) analysis of lipoproteins and lipids of the types of INSTI and NNRTI in the discovery cohort. X-axis shows PC1, Y-axis shows PC2.

A: All types of INSTI showed considerable overlap in PC analysis, showing homogeneity between lipoproteins and lipids between the different INSTI types.

B: There were considerable differences between the different types of NNRTI in PC analysis, showing heterogeneity in lipoproteins and lipids between the different NNRTI types.

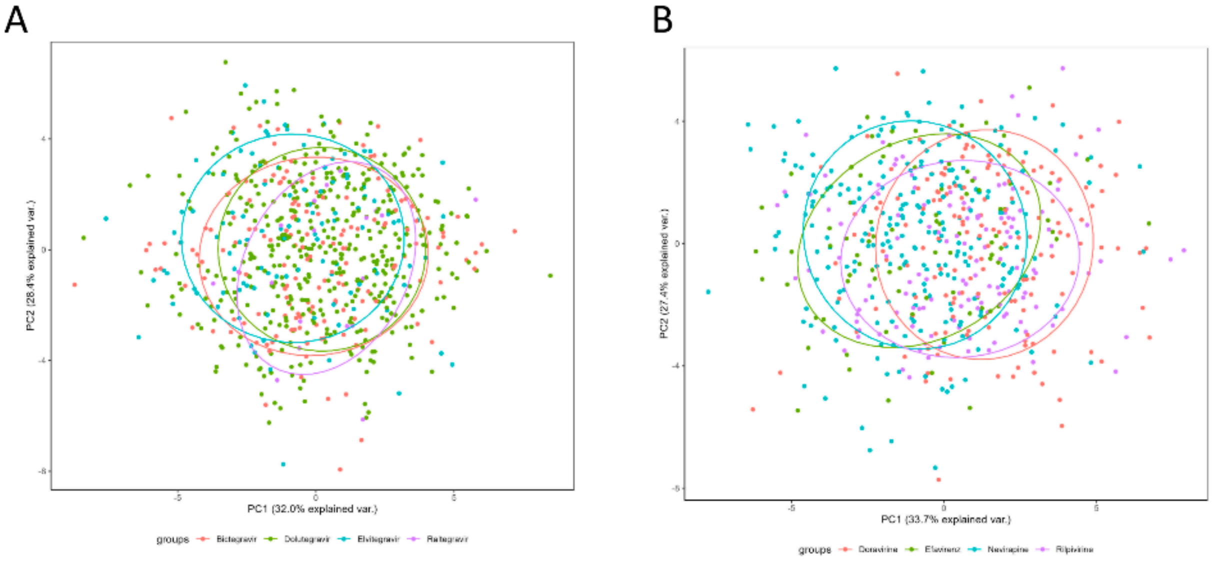

Supplementary table S3: Baseline characteristics of INSTI, NNRTI nevirapine and NNRTI doravirine users in the discovery (A) and validation cohort (B).

Supplementary table S3A:

| | Discovery cohort | | | |
|---|---|---|---|---|
| | **INSTI** 730 patients | **Nevirapine** 236 patients | **Doravirine** 164 patients | P-val |
| **Age in years (IQR)** | 51.0 (41.0 - 58.0) | 55.0 (49.8 - 63.0) | 50.0 (41.0 - 57.2) | < 0.0001 |
| **Sex at birth (male)** | 619 (84.8%) | 202 (85.6%) | 141 (86.0%) | 0.94 |
| **BMI in kg/m2 Median (IQR)** | 25.0 (22.7 - 27.7) | 24.5 (22.2 - 27.3) | 24.9 (22.4 - 28.0) | 0.20 |
| **Ethnicity (white)** | 543 (74.4%) | 192 (81.4%) | 115 (70.1%) | 0.025 |
| **HIV duration in years Median (IQR)** | 10.8 (5.7 - 16.5) | 19.0 (13.1 - 23.3) | 11.6 (6.7 - 15.5) | < 0.0001 |
| **cART Duration in years Median (IQR)** | 8.3 (4.7 - 13.4) | 16.1 (10.4 - 21.6) | 9.5 (5.8 - 13.1) | < 0.0001 |
| **Latest CD4 count ($x10^6$ cells/L) Median (IQR)** | 740.0 (550.5 - 940.0) | 673.0 (530.0 - 845.0) | 707.5 (553.2 - 941.8) | 0.01 |
| **CD4 Nadir ($x10^6$ cells/L) Median (IQR)** | 280.0 (160.0 - 422.5) | 230.0 (120.0 - 310.0) | 285.0 (164.8 - 413.8) | < 0.0001 |
| **Viral Load Zenith (copies/ml) Median (IQR)** | 97000.0 (36650.5 - 248154.0) | 100000.0 (49300.0 - 261000.0) | 102347.0 (37075.0 - 300000.0) | 0.27 |
| **Currently smoking** | 238 (32.6%) | 60 (25.4%) | 38 (23.2%) | 0.01 |
| Missing | 56 (7.7%) | 12 (5.1%) | 14 (8.5%) | |
| **Packyears Median (IQR)** | 6.0 (0.0 - 22.0) | 11.8 (0.0 - 27.9) | 0.3 (0.0 - 15.4) | 0.0002 |
| **Had Non-AIDS malignancy** | 28 (3.8%) | 18 (7.6%) | 6 (3.7%) | 0.060 |
| **Had previous cardiovascular disease** | 214 (29.3%) | 98 (41.5%) | 41 (25.0%) | 0.0004 |
| **On lipid lowering medication** | 141 (19.3%) | 53 (22.5%) | 22 (13.4%) | 0.070 |

Supplementary table S3B:

| | Validation cohort | | | |
|---|---|---|---|---|
| | **INSTI** | **Nevirapine** | **Doravirine** | P-val |
| | **209 patients** | **38 patients** | **5 patients** | |
| **Age in years (IQR)** | 52.0 (45.0 - 61.0) | 54.5 (48.0 - 59.8) | 48.0 (37.0 - 54.0) | 0.34 |
| **Sex at birth (male)** | 178 (85.2%) | 32 (84.2%) | 4 (80.0%) | 0.76 |
| **BMI in kg/m2 Median (IQR)** | 25.8 (23.0 - 28.4) | 25.4 (22.3 - 27.3) | 28.2 (24.8 - 28.3) | 0.75 |
| **Ethnicity (white)** | 180 (86.1%) | 33 (86.8%) | 4 (80.0%) | 0.74 |
| **HIV duration in years Median (IQR)** | 7.0 (3.5 - 12.7) | 15.7 (11.7 - 22.3) | 10.1 (3.3 - 10.5) | < 0.0001 |
| **cART Duration in years Median (IQR)** | 6.0 (3.3 - 10.6) | 12.9 (9.2 - 21.3) | 10.0 (3.3 - 10.4) | < 0.0001 |
| **Latest CD4 count ($x10^6$ cells/L) Median (IQR)** | 660.0 (480.0 - 810.0) | 660.0 (462.5 - 800.0) | 700.0 (530.0 - 950.0) | 0.73 |
| **CD4 Nadir ($x10^6$ cells/L) Median (IQR)** | 290.0 (170.0 - 452.5) | 280.0 (190.0 - 330.0) | 300.0 (130.0 - 420.0) | 0.18 |
| **Viral Load Zenith (copies/ml) Median (IQR)** | 156748.0 (39240.8 - 346862.5) | 100000.0 (46000.0 - 223214.0) | 973984.0 (606326.0 - 3675318.0) | 0.005 |
| **Currently smoking** | 59 (28.2%) | 15 (39.5%) | 1 (20.0%) | 0.33 |
| Missing | 23 (11.0%) | 4 (10.5%) | 1 (20.0%) | |
| **Packyears Median (IQR)** | 6.0 (0.0 - 28.5) | 12.5 (0.0 - 36.0) | 0.0 (0.0 - 11.0) | 0.61 |
| **Had Non-AIDS malignancy** | 12 (5.7%) | 2 (5.3%) | 0 (0.0%) | 1.0 |
| **Had previous cardiovascular disease** | 66 (31.6%) | 18 (47.4%) | 1 (20.0%) | 0.13 |
| **On lipid lowering medication** | 40 (19.1%) | 14 (36.8%) | 0 (0.0%) | 0.029 |
